# Composite Indices Using 3 or 4 Components of the Core Data Set Have Similar Predictive Ability to Measure Disease Activity in RA: Evidence from the DANCER and REFLEX Studies

**DOI:** 10.1155/2013/367190

**Published:** 2013-12-04

**Authors:** Martin J. Bergman, William Reiss, Carol Chung, Pamela Wong, Adam Turpcu

**Affiliations:** ^1^Rheumatology Department, Drexel University College of Medicine and Taylor Hospital, 8 Morton Avenue, Suite 304, Ridley Park, Philadelphia, PA 19078-2216, USA; ^2^Genentech, Inc., 1 DNA Way, South San Francisco, CA 94080, USA

## Abstract

*Background*. Understanding how disease-assessment indices perform in rheumatoid arthritis (RA) clinical trials can inform their use in routine practice. The study objective was to assess the capacity of combinations of RA Core Data Set measures to distinguish rituximab from control treatment. *Methods*. Post hoc analysis of two randomised clinical trials was used. Composite Efficacy Indices were derived by combining three or four RA Core Data Set measures from three possible sources: physician, patient, and laboratory. *Results*. All 105 Composite Efficacy Indices evaluated significantly distinguished rituximab from control treatment (*P* < 10^−7^). Generally, indices containing measures from three different sources had a greater capacity to distinguish rituximab from control treatment than indices containing three measures from one source. Composite Efficacy Indices performed as well as validated indices such as DAS28, RAPID3, and CDAI. *Conclusions*. All indices composed of three or four RA Core Data Set measures have a similar capacity to detect treatment differences. These results suggest that the precise measurement used is less important than whether any measurement is performed, although selection should be consistent for each patient. Therefore, the choice of assessment tool should not be limited to a prescribed list and should instead be left to the clinician's discretion.

## 1. Introduction

In an effort to improve patient outcomes, recent consensus guidelines have recommended treating patients with rheumatoid arthritis (RA) to a target of clinical remission [1]. To accomplish this aim, different measurement tools of disease activity have been developed. Recently, the American College of Rheumatology (ACR) has published guidelines listing “preferred” indices to be used in clinical practice, including Disease Activity Score in 28 joints (DAS28), Simplified Disease Activity Index (SDAI), Clinical Disease Activity Index (CDAI), and Routine Assessment of Patient Index Data 3 (RAPID3) [2].

These recommended indices are derived from the ACR RA Core Data Set measures [3] and include data from three sources: (1) health professional: assessor global (DOCGL), tender joint count (TJC), and swollen joint count (SJC); (2) patient questionnaires: patient global estimate (PATGL), pain, and physical function (FN); and (3) laboratory tests: C-reactive protein (CRP) level or erythrocyte sedimentation rate (ESR). Each of the recommended indices has variable complexities, which may serve as a barrier to use in clinical practice. Despite these differences, moderate-to-strong levels of agreement have been observed between the indices [4, 5]. Given this agreement, it is speculated that other indices composed of different combinations of RA Core Data Set measures could also be of use in assessing disease activity.

The objective of this study was to determine whether composite indices of any three or four RA Core Data Set measures, not just the “recommended” indices, have a similar capacity to distinguish rituximab from control treatment.

## 2. Methods

**Figure 1:**
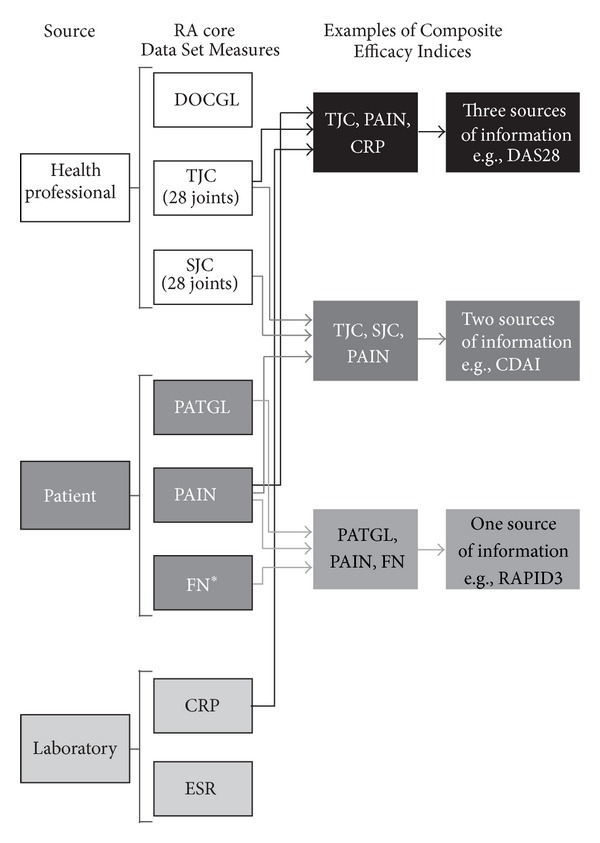
Combinations of RA Core Data Set measures used to derive Composite Efficacy Indices. *Measured using the Health Assessment Questionnaire. For comparison, DAS28, CDAI, and RAPID3 are derived using different formulae. DAS28 includes 4 RA Core Data Set measures (TJC, SJC, CRP (or ESR), and PATGL) from three sources. CDAI includes four RA Core Data Set measures (TJC, SJC, DOCGL, and PATGL) from two sources. RAPID3 includes three RA Core Data Set measures (PATGL, PAIN, and FN) from one source. CDAI: Clinical Disease Activity Index; CRP: C-reactive protein; DAS28: Disease Activity Score in 28 joints; DOCGL: assessor global; ESR: erythrocyte sedimentation rate; FN: physical function; PAIN: pain; PATGL: patient global estimate; RAPID3: Routine Assessment of Patient Index Data 3; SJC: swollen joint count; TJC: tender joint count.

Patient data from DANCER, a phase IIb study comparing placebo and two doses of rituximab in RA patients who had an inadequate response to methotrexate (MTX) and 1–5 other disease-modifying antirheumatic drugs or biologicals, and REFLEX, a phase III study comparing placebo and rituximab in RA patients with an inadequate response to one or more tumor necrosis factor inhibitors, were used to develop different composite indices [6, 7]. Composite Efficacy Indices were derived by combining 3 or 4 RA Core Data Set measures from 3 possible sources, health professional evaluation, patient questionnaires, and laboratory tests (Figure 1), and no more than one laboratory test (either CRP or ESR). Laboratory test values were log transformed prior to rescaling. Analyses were limited to the approved rituximab dose (2 × 1000 mg) group and placebo group intent-to-treat populations. All RA Core Data Set measures were rescaled from 0–10 and were equally weighted in each possible combination. For each combination, changes from baseline to the last observation on or before week 24 were compared between rituximab and placebo treatment using Kruskal-Wallis tests. Standardized response means (SRMs) were used to estimate a Composite Efficacy Index's ability to distinguish between responsiveness to rituximab and responsiveness to placebo and were calculated [8] using the following formula:

(1) SRM=(mRTX−mPlacebo) ×(((nRTX−1)sRTX2+(nPlacebo−1)sPlacebo2)(nRTX+nPlacebo−2))−1,

where *m* is mean; *n* is number of patients; RTX is rituximab; *s* is standard deviation of change scores from baseline.

## 3. Results

In general, demographics and clinical characteristics were balanced across both treatment groups and trials and have been described elsewhere [6, 7]. Baseline characteristics of key efficacy indices and RA Core Data Set measures are given in Table 1.

**Figure 2:**
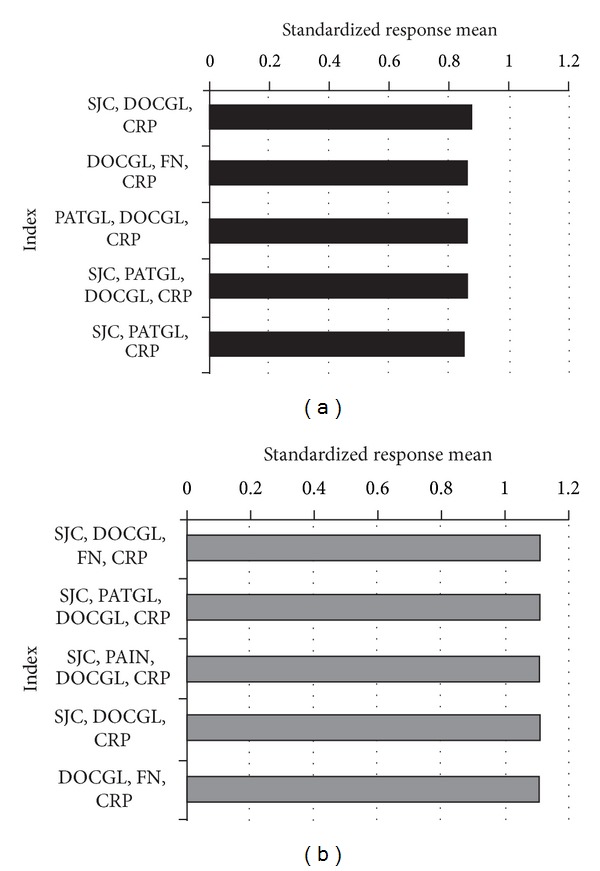
Indices with the greatest standardized response means in (a) DANCER (95% confidence interval for each index was ±0.22. SRMs: DAS28 = 0.77, CDAI = 0.66, and RAPID3 = 0.60) and (b) REFLEX (95% confidence interval for each index was ±0.18. SRMs: DAS28 = 1.04, CDAI = 0.92, and RAPID3 = 0.83). CRP: C-reactive protein; DOCGL: assessor global; FN: physical function; PATGL: patient global estimate; SJC: swollen joint count.

A total of 105 Composite Efficacy Indices, or the maximum number of possible combinations with 3 or 4 Core Data Set measures, were evaluated (Table 2). All indices were found to significantly distinguish rituximab from control treatment. In DANCER, *P* values ranged from 7 × 10^−7^ to 5 × 10^−13^ for three-measure indices and from 2 × 10^−7^ to 2 × 10^−12^ for four-measure indices. In REFLEX, *P* values for three- and four-measure indices ranged from 1 × 10^−17^ to 2 × 10^−28^ and 9 × 10^−20^ to 3 × 10^−28^, respectively. Generally, indices containing measures from three different sources had a greater capacity to distinguish rituximab from control treatment than indices containing three measures from one source. Indices showing the greatest SRMs are shown in Figure 2. The best performing index in DANCER (SRM 0.87 (95% CI, 0.65, 1.09)) comprised three measures: SJC, DOCGL, and CRP. In REFLEX, two indices of four measures each performed equally well (SRM 1.13 (95% CI, 0.95, 1.31)): SJC, DOCGL, FN, and CRP and SJC, PATGL, DOCGL, and CRP.

## 4. Discussion

A number of validated and nonvalidated indices are available to assess RA disease status. Identifying those indices that can accurately measure disease activity while requiring less time and resources would be desirable from both physician and patient perspectives. The results of our analysis indicate that any index comprising any three or four RA Core Data Set measures was capable of distinguishing rituximab from control treatment at highly statistically significant levels. Furthermore, the Composite Efficacy Indices performed well in comparison to validated indices when assessed by SRM.

The best performing indices were those that included both physician- and laboratory-derived measures suggesting that there may be additional value in including data from multiple domains. However, laboratory results are often unavailable at the time of patient assessment. When using indices that include laboratory tests in a practice setting, immediate calculation of disease activity scores is not always possible. A further consideration is physician resources, particularly the assessment of joint counts, which can be time consuming for the physician [9]. Based on the results of this study, insistence on the inclusion of specific measures, such as TJC or SJC, does not appear to be supported. In fact, a number of 3-component measures without a formal tender or swollen count (e.g., PATGL, DOCGL, and CRP) had better discriminatory value in differentiating rituximab from control treatment (*P* = 2 × 10^−27^ and 2 × 10^−12^ in REFLEX and DANCER, resp.) than that of a current “gold standard,” CDAI (*P* = 8 × 10^−23^ and 4 × 10^−9^ in REFLEX and DANCER, resp.). The clinical importance of such small differences is questionable as even the “worst” measure, RAPID3 (PAIN, PATGL, and FN), had *P* values significantly below the thresholds that are commonly reported in the medical literature (*P* = 1 × 10^−17^ and 7 × 10^−7^ in REFLEX and DANCER, resp.). The effectiveness of patient-derived indices may therefore be worthy of consideration.

## 5. Conclusions

In conclusion, these results suggest that any index using three or four measures from the RA Core Data Set is capable of distinguishing active from control treatment. While certain measurements have been proposed to be preferred, they are not superior to other measures currently in development or in use. Based on our data, it would appear that the precise measurement used may be less important than whether any measurement is performed. While more studies are needed to validate these findings, our results suggest that the choice of measurement tool should not be limited to a prescribed list of “better” or “approved” tools and may instead be left to the discretion of the clinician, allowing for the flexibility to tailor disease activity assessments according to point-of-care time and resource limitations.

Taken together, the results of this study suggest that Composite Efficacy Indices comprised of any combination of three or four measures from the RA Core Data Set perform well in discriminating between treatment responses to rituximab and placebo.

## Figures and Tables

**Table 1:** Patients' baseline characteristics for key efficacy measurements and rheumatoid arthritis Core Data Set measures in the DANCER [6] and REFLEX [7] clinical trials.

Mean (SD)	REFLEX		DANCER	
	Placebo (*n* = 201)	RTX (*n* = 298)	Placebo (*n* = 143)	RTX (*n* = 185)
DAS28-ESR (0–10)	6.8 (0.9)	6.9 (1.0)	6.8 (0.8)	6.7 (0.9)
DAS28 (3)-ESR (0–10)	6.5 (0.9)	6.5 (0.9)	6.5 (0.7)	6.4 (0.8)
CDAI (0–76)	44.6 (13.2)	45.9 (13.7)	45.9 (11.6)	43.8 (12.7)
RAPID3 (0–10)	5.8 (1.7)	5.7 (1.8)	5.4 (1.6)	5.3 (1.7)
DOCGL (0–10)	6.7 (1.6)	6.9 (1.6)	6.6 (1.5)	6.6 (1.6)
TJC (0–28)	16.5 (7.0)	17.2 (7.1)	18.5 (5.9)	17.4 (6.5)
SJC (0–28)	14.4 (5.9)	14.9 (5.9)	14.2 (5.3)	13.2 (5.7)
PATGL (0–10)	7.0 (2.0)	6.9 (2.1)	6.6 (1.9)	6.6 (2.0)
PAIN (0–10)	6.4 (2.1)	6.4 (2.2)	6.0 (1.9)	5.9 (2.0)
FN (HAQ) (0–3)	1.9 (0.5)*	1.9 (0.6)	1.7 (0.6)	1.7 (0.6)
CRP (mg/dL)	3.8 (4.1)	3.7 (3.8)	3.1 (3.0)	3.0 (3.5)
ESR (mm/h)	48.4 (27.7)	47.9 (25.6)	39.7 (20.8)	42.0 (23.3)

*n = 200. CDAI: Clinical Disease Activity Index; CRP: C-reactive protein; DAS28: Disease Activity Score in 28 joints; DAS28 (3): Disease Activity Score in 28 joints excluding patient global health component; DOCGL: assessor global; ESR: erythrocyte sedimentation rate; FN: physical function; HAQ: Health Assessment Questionnaire; PAIN: pain; PATGL: patient global estimate; RAPID3: Routine Assessment of Patient Index Data 3; RTX: rituximab; SD: standard deviation; SJC: swollen joint count; TJC: tender joint count.

**Table 2:** Treatment comparisons of changes from baseline in composite indices by number of components and RA Core Data Set measures.

Components	Number of components	REFLEX		DANCER	
		SRM	*P* value	SRM	*P* value
SJC, DOCGL, FN, CRP	4	1.13	3 × 10^−28^	0.85	2 × 10^−12^
SJC, PATGL, DOCGL, CRP	4	1.13	4 × 10^−28^	0.86	4 × 10^−12^
SJC, PAIN, DOCGL, CRP	4	1.12	5 × 10^−28^	0.84	7 × 10^−12^
SJC, PATGL, FN, CRP	4	1.09	1 × 10^−27^	0.82	2 × 10^−11^
SJC, PAIN, FN, CRP	4	1.09	1 × 10^−27^	0.81	3 × 10^−11^
TJC, DOCGL, FN, CRP	4	1.10	3 × 10^−27^	0.81	2 × 10^−11^
PATGL, DOCGL, FN, CRP	4	1.09	6 × 10^−27^	0.83	8 × 10^−12^
PAIN, DOCGL, FN, CRP	4	1.09	7 × 10^−27^	0.82	1 × 10^−11^
TJC, PAIN, DOCGL, CRP	4	1.09	1 × 10^−26^	0.80	6 × 10^−11^
TJC, PATGL, DOCGL, CRP	4	1.10	1 × 10^−26^	0.81	2 × 10^−11^
SJC, TJC, FN, CRP	4	1.07	1 × 10^−26^	0.77	1 × 10^−10^
SJC, TJC, PATGL, CRP	4	1.07	2 × 10^−26^	0.79	5 × 10^−11^
SJC, TJC, DOCGL, CRP	4	1.08	2 × 10^−26^	0.80	2 × 10^−11^
SJC, TJC, PAIN, CRP	4	1.07	3 × 10^−26^	0.77	2 × 10^−10^
TJC, PATGL, FN, CRP	4	1.06	4 × 10^−26^	0.77	2 × 10^−10^
SJC, PAIN, PATGL, CRP	4	1.05	5 × 10^−26^	0.79	3 × 10^−10^
TJC, PAIN, FN, CRP	4	1.06	7 × 10^−26^	0.76	3 × 10^−10^
PAIN, PATGL, DOCGL, CRP	4	1.06	2 × 10^−25^	0.79	1 × 10^−10^
SJC, PAIN, DOCGL, ESR	4	1.05	3 × 10^−25^	0.79	4 × 10^−11^
SJC, PATGL, DOCGL, ESR	4	1.05	3 × 10^−25^	0.81	3 × 10^−11^
SJC, DOCGL, FN, ESR	4	1.04	3 × 10^−25^	0.80	2 × 10^−11^
TJC, PAIN, PATGL, CRP	4	1.03	1 × 10^−24^	0.75	2 × 10^−9^
SJC, PAIN, FN, ESR	4	1.01	2 × 10^−24^	0.75	2 × 10^−10^
TJC, DOCGL, FN, ESR	4	1.02	3 × 10^−24^	0.75	2 × 10^−10^
SJC, PATGL, FN, ESR	4	1.01	4 × 10^−24^	0.77	2 × 10^−10^
TJC, PATGL, DOCGL, ESR	4	1.02	4 × 10^−24^	0.76	4 × 10^−10^
TJC, PAIN, DOCGL, ESR	4	1.02	7 × 10^−24^	0.75	5 × 10^−10^
SJC, TJC, FN, ESR	4	0.99	7 × 10^−24^	0.72	7 × 10^−10^
SJC, TJC, DOCGL, ESR	4	1.00	9 × 10^−24^	0.75	2 × 10^−10^
SJC, TJC, PATGL, ESR	4	1.00	9 × 10^−24^	0.74	9 × 10^−10^
SJC, TJC, PAIN, ESR	4	1.00	9 × 10^−24^	0.72	2 × 10^−9^
PATGL, DOCGL, FN, ESR	4	1.01	2 × 10^−23^	0.78	8 × 10^−11^
PAIN, DOCGL, FN, ESR	4	1.01	2 × 10^−23^	0.77	2 × 10^−10^
SJC, PAIN, DOCGL, FN	4	0.99	2 × 10^−23^	0.72	3 × 10^−9^
SJC, PAIN, PATGL, ESR	4	0.98	2 × 10^−23^	0.74	2 × 10^−9^
SJC, PATGL, DOCGL, FN	4	0.99	3 × 10^−23^	0.73	2 × 10^−9^
PAIN, PATGL, FN, CRP	4	0.98	4 × 10^−23^	0.74	2 × 10^−9^
SJC, TJC, PAIN, DOCGL	4	0.97	6 × 10^−23^	0.69	8 × 10^−9^
SJC, TJC, PATGL, DOCGL	4	0.97	8 × 10^−23^	0.71	4 × 10^−9^
TJC, PAIN, FN, ESR	4	0.98	1 × 10^−22^	0.71	5 × 10^−9^
TJC, PATGL, FN, ESR	4	0.98	1 × 10^−22^	0.72	3 × 10^−9^
SJC, PAIN, PATGL, DOCGL	4	0.97	1 × 10^−22^	0.71	6 × 10^−9^
SJC, TJC, DOCGL, FN	4	0.96	2 × 10^−22^	0.69	7 × 10^−9^
PAIN, PATGL, DOCGL, ESR	4	0.98	2 × 10^−22^	0.75	1 × 10^−9^
TJC, PAIN, DOCGL, FN	4	0.96	4 × 10^−22^	0.68	8 × 10^−9^
SJC, TJC, PAIN, FN	4	0.94	4 × 10^−22^	0.66	4 × 10^−8^
TJC, PATGL, DOCGL, FN	4	0.96	4 × 10^−22^	0.70	6 × 10^−9^
SJC, TJC, PATGL, FN	4	0.94	7 × 10^−22^	0.67	3 × 10^−8^
TJC, PAIN, PATGL, ESR	4	0.96	1 × 10^−21^	0.70	2 × 10^−8^
SJC, TJC, PAIN, PATGL	4	0.93	1 × 10^−21^	0.66	7 × 10^−8^
TJC, PAIN, PATGL, DOCGL	4	0.95	2 × 10^−21^	0.68	3 × 10^−8^
SJC, PAIN, PATGL, FN	4	0.92	3 × 10^−21^	0.67	4 × 10^−8^
PAIN, PATGL, DOCGL, FN	4	0.92	1 × 10^−20^	0.68	2 × 10^−8^
TJC, PAIN, PATGL, FN	4	0.90	3 × 10^−20^	0.63	2 × 10^−7^
PAIN, PATGL, FN, ESR	4	0.90	9 × 10^−20^	0.68	2 × 10^−8^
SJC, FN, CRP	3	1.11	2 × 10^−28^	0.84	3 × 10^−12^
DOCGL, FN, CRP	3	1.12	2 × 10^−28^	0.86	5 × 10^−13^
SJC, PATGL, CRP	3	1.11	3 × 10^−28^	0.85	7 × 10^−12^
SJC, DOCGL, CRP	3	1.12	4 × 10^−28^	0.87	8 × 10^−13^
SJC, PAIN, CRP	3	1.10	8 × 10^−28^	0.83	2 × 10^−11^
PATGL, DOCGL, CRP	3	1.11	2 × 10^−27^	0.86	2 × 10^−12^
PAIN, DOCGL, CRP	3	1.11	4 × 10^−27^	0.84	7 × 10^−12^
TJC, DOCGL, CRP	3	1.10	4 × 10^−27^	0.82	1 × 10^−11^
TJC, FN, CRP	3	1.07	6 × 10^−27^	0.78	9 × 10^−11^
TJC, PATGL, CRP	3	1.07	4 × 10^−26^	0.79	1 × 10^−10^
SJC, TJC, CRP	3	1.05	9 × 10^−26^	0.77	7 × 10^−11^
TJC, PAIN, CRP	3	1.07	1 × 10^−25^	0.77	5 × 10^−10^
PATGL, FN, CRP	3	1.03	5 × 10^−25^	0.79	7 × 10^−11^
PAIN, FN, CRP	3	1.03	6 × 10^−25^	0.77	2 × 10^−10^
SJC, FN, ESR	3	1.00	1 × 10^−24^	0.77	2 × 10^−11^
SJC, DOCGL, ESR	3	1.02	2 × 10^−24^	0.80	1 × 10^−11^
SJC, PAIN, ESR	3	1.01	2 × 10^−24^	0.76	1 × 10^−10^
SJC, PATGL, ESR	3	1.01	3 × 10^−24^	0.79	4 × 10^−11^
DOCGL, FN, ESR	3	1.00	6 × 10^−24^	0.80	1 × 10^−11^
PATGL, DOCGL, ESR	3	1.02	1 × 10^−23^	0.80	4 × 10^−11^
PAIN, DOCGL, ESR	3	1.01	2 × 10^−23^	0.78	1 × 10^−10^
TJC, DOCGL, ESR	3	1.00	2 × 10^−23^	0.75	1 × 10^−10^
SJC, PAIN, DOCGL	3	0.99	3 × 10^−23^	0.72	3 × 10^−9^
PAIN, PATGL, CRP	3	0.99	3 × 10^−23^	0.74	2 × 10^−9^
SJC, PATGL, DOCGL	3	0.98	3 × 10^−23^	0.74	1 × 10^−9^
SJC, DOCGL, FN	3	0.97	1 × 10^−22^	0.72	2 × 10^−9^
TJC, FN, ESR	3	0.96	1 × 10^−22^	0.71	1 × 10^−9^
TJC, PATGL, ESR	3	0.98	1 × 10^−22^	0.73	3 × 10^−9^
SJC, TJC, ESR	3	0.95	3 × 10^−22^	0.70	1 × 10^−9^
TJC, PAIN, ESR	3	0.98	3 × 10^−22^	0.70	6 × 10^−9^
SJC, PAIN, FN	3	0.94	5 × 10^−22^	0.66	3 × 10^−8^
TJC, PATGL, DOCGL	3	0.95	7 × 10^−22^	0.70	7 × 10^−9^
TJC, PAIN, DOCGL	3	0.95	7 × 10^−22^	0.68	2 × 10^−8^
TJC, DOCGL, FN	3	0.94	9 × 10^−22^	0.68	1 × 10^−8^
SJC, PATGL, FN	3	0.93	1 × 10^−21^	0.68	2 × 10^−8^
SJC, TJC, PATGL	3	0.92	2 × 10^−21^	0.66	4 × 10^−8^
SJC, TJC, PAIN	3	0.92	2 × 10^−21^	0.64	8 × 10^−8^
SJC, TJC, DOCGL	3	0.93	2 × 10^−21^	0.67	1 × 10^−8^
PAIN, DOCGL, FN	3	0.94	2 × 10^−21^	0.69	9 × 10^−9^
PATGL, DOCGL, FN	3	0.94	4 × 10^−21^	0.70	6 × 10^−9^
SJC, PAIN, PATGL	3	0.91	6 × 10^−21^	0.66	1 × 10^−7^
SJC, TJC, FN	3	0.90	1 × 10^−20^	0.63	1 × 10^−7^
PATGL, FN, ESR	3	0.92	1 × 10^−20^	0.72	1 × 10^−9^
PAIN, FN, ESR	3	0.92	1 × 10^−20^	0.71	3 × 10^−9^
TJC, PAIN, FN	3	0.90	2 × 10^−20^	0.62	3 × 10^−7^
PAIN, PATGL, DOCGL	3	0.92	2 × 10^−20^	0.68	3 × 10^−8^
TJC, PATGL, FN	3	0.89	3 × 10^−20^	0.64	1 × 10^−7^
TJC, PAIN, PATGL	3	0.88	1 × 10^−19^	0.62	6 × 10^−7^
PAIN, PATGL, ESR	3	0.89	1 × 10^−19^	0.68	6 × 10^−8^
PAIN, PATGL, FN	3	0.83	1 × 10^−17^	0.60	7 × 10^−7^

SRMs: REFLEX: DAS28 = 1.04, CDAI = 0.92, and RAPID3 = 0.83; DANCER: DAS28 = 0.77, CDAI = 0.66, and RAPID3 = 0.6. Kruskal-Wallis *P* values compare the changes from baseline to the last observation on or before week 24 between rituximab and placebo treatment. These *P*-values were not adjusted for multiplicity. CRP: C-reactive protein; DOCGL: assessor global; ESR: erythrocyte sedimentation rate; FN: physical function; PAIN: pain; PATGL: patient global estimate; SJC: swollen joint count; TJC: tender joint count.
